# Social origins, biological treatments: The public health implications of common mental disorders in India

**DOI:** 10.4103/0019-5545.46068

**Published:** 2005

**Authors:** Vikram Patel

**Affiliations:** * Reader in International Mental Health London School of Hygiene and Tropical Medicine, London, UK

**Keywords:** Common mental disorders, biological treatment, depression, anxiety

## Abstract

Common mental disorders (CMD) is a term used to describe depressive and anxiety disorders. It replaces the old term ‘neuroses’ and is widely used because of the high level of co-morbidity of depression and anxiety, which limits the validity of categorical models of classification of neurotic disorders, particularly in primary care settings. The global public health significance of CMD is highlighted by the fact that in developing countries, depression is the leading cause of years lived with disability in both men and women aged 15–44 years. This oration brings together research evidence, mostly from South Asia, to show that although the aetiology of CMD may lie in the socioeconomic circumstances faced by many patients, biological treatments such as antidepressants may be among the most cost-effective treatments in resource-poor settings. The oration demonstrates the public health implications of CMD by briefly reviewing the burden of CMD in the region and presents evidence linking the risk for CMD associated with two of the region's most important public health risk factors—poverty and gender disadvantage. The oration also presents recent evidence to establish the association of CMD with some of the region's most important public health issues: maternal and child health; and reproductive and sexual health. Next, the evidence for the efficacy of treatments for CMD in developing countries is presented, focusing on a series of recent trials that show that both psychosocial and biological treatments are effective. Finally, the implications for policy and future research are considered.

## INTRODUCTION

Common mental disorders (CMD) are depressive and anxiety disorders that are typically encountered in community and primary care settings.^1^ Although depressive and anxiety disorders are classified as separate diagnostic categories in ICD-10,^2^ the concept of CMD is acknowledged as being more valid for public health interventions due to the high degree of co-morbidity between subcategories and the similarity in epidemiological profiles and treatment responsiveness.^1^,^3^–^5^ In the South-East Asian region, 11% of disability-adjusted life-years (DALYs) and 27% of years lived with disability (YLD) are attributed to neuropsychiatric disease. Depressive disorders are the most important neuropsychiatric cause of disease burden.^6^ CMD lead to profound levels of disability through symptoms such as tiredness and sleep problems, and are associated with increased healthcare costs and reduced economic productivity.^7^–^9^ A review of 8 epidemiological studies on CMD in South Asia shows that the prevalence in primary care was 26.3% (95% CI 25.3%—27.4%).^10^ In a study done in Goa, the rate of CMD was 46.5% in adult primary care attenders.^11^ Patients with CMD spent twice the number of days in the previous month being unable to work as usual due to their illness. Over half the cases in primary care remain chronic for up to 12 months.^12^

Clinical studies show that while somatic symptoms are the commonest presenting complaints, psychological and cognitive symptoms can be elicited in the majority of patients on inquiry.^13^ Most patients and general health workers do not view CMD as being psychiatric or mental disorders, which may partly explain the relatively low recognition rates for CMD in primary care. Instead, psychosocial and spiritual models of illness causation and management are often preferred.^13^–^15^ The clinical validity of the subcategories of CMD is in doubt; in primary care, a dimensional model of distress may be easier and more practical to use.^3^ The majority of patients do not receive evidence-based treatments; typically, symptomatic treatments are provided (for example, vitamins and tonics for the complaint of fatigue, hypnotics for sleep difficulties, etc.). It is not surprising that surveys of prescription behaviour in India show that a majority of drugs used are of ‘doubtful value’.^16^ Inappropriate treatment is associated with chronicity, disability and increased healthcare costs.

Despite this substantial body of epidemiological evidence demonstrating the considerable burden of CMD in developing countries, CMD remain a low priority in public health. In this oration, I will argue that CMD are a high priority in public health, using evidence on the links between CMD and established public policy priorities, and evidence that CMD can be treated effectively using locally available and affordable treatments.

## POVERTY, GENDER DISADVANTAGE AND CMD

Diseases that disproportionately affect the poor, or women who face disadvantages on account of their gender, are prioritized by public health policy-makers. Stressful life experiences, such as exposure to violence and poor physical health, which are well-recognized risk factors for mental disorders, are more likely to be experienced by poor people. Thus, it is not surprising that virtually all population-based studies of the risk factors for mental disorders, particularly depressive and anxiety disorders, consistently show that the poor and marginalized are at greater risk of suffering from these.^8^ We also know that mental disorders impoverish people, both due to the increased costs of healthcare—often sought through private providers—and lost employment opportunities. Most mental illnesses are relatively simple and cheap to treat, and evidence from clinical trials shows that efficacious treatment is associated with significant reductions in overall healthcare costs.^17^ Thus, treating mental disorders, particularly in the poor who bear the disproportionate burden of suffering, would help them work more productively and reduce their healthcare expenditure, facilitating the conditions necessary to rise out of poverty. One of the most consistent risk factors for CMD is female sex; this increased risk has also been replicated in developing countries.^18^ A recent review on the possible explanations for these sex differences found no evidence to support a hormonal or other biological mechanism.^19^ On the other hand, there is growing evidence that gender disadvantage, as indicated by exposure to intimate partner violence and low levels of autonomy in decision-making, are key risk factors for CMD in women.^20^

## CMD AND MATERNAL AND CHILD HEALTH

Maternal and child health is one of India's most important public health priorities. One of the commonest health problems affecting mothers during pregnancy and after childbirth is depression. A large number of studies from most regions of the developing world show that between 10% and 30% of mothers will suffer from depression.^21^–^23^ Depressed mothers are much more disabled and less likely to take care of their needs. Suicide is a leading cause of maternal death in developed countries.^24^ Suicide is now a leading cause of death among young women in the reproductive age group in the world's two most populous countries, India and China.^25^,^26^ It is plausible that depression in mothers may also lead to increased maternal mortality, both through adversely impacting on their physical health needs, as well as more directly through suicide. A series of studies from South Asia have demonstrated that early childhood failure to thrive, as indicated by undernutrition and stunting in under-1-year-old babies, is independently associated with depression in mothers.^27^ For example, a recent population-based cohort study from Pakistan has shown that babies of mothers who were depressed during pregnancy and in the postnatal period were more than 5 times at greater risk for being underweight and stunted at 6 months than babies of non-depressed mothers, even after adjustment for other known confounders such as maternal socioeconomic status.^28^ Childhood failure to thrive is a major risk factor for child mortality and thus, it would be plausible to hypothesize that depression in mothers is also associated with increased child mortality. Indeed, evidence shows that depressed mothers are more likely to cease breastfeeding, and their babies are significantly more likely to suffer from diarrhoeal episodes or to not have their complete immunization, all of these being recognized risk factors for childhood mortality.^28^ This study also showed that depression during pregnancy was strongly associated with low birth weight, an association that has been replicated in studies in India and Brazil (personal communication).

## CMD AND REPRODUCTIVE AND SEXUAL HEALTH

Among the most common complaints in women are those related to their reproductive and sexual health, notably complaints of abnormal vaginal discharge and fatigue.^29^ Not surprisingly, such complaints are the focus of reproductive health programmes in the country. These complaints have typically been assumed to be the result of poor reproductive health; the complaint of vaginal discharge, for example, is attributed to reproductive tract infections, while the complaint of fatigue is attributed to anaemia. However, a growing body of evidence has demonstrated that these assumptions are incorrect; for example, a study in Bangladesh found that only 1 in 3 women with the complaint of abnormal vaginal discharge in fact had any infection.^30^ A population-based cohort study of nearly 2500 women has recently been completed in Goa, India; its objective was to investigate psychosocial aetiologies for these complaints, focusing on mental health and gender disadvantage. The rationale for these hypotheses was that poor mental health was a recognized risk factor for medically unexplained symptoms in developed countries and was a recognized risk factor for sexual complaints among men in South Asia (the *dhat* syndrome).^31^ These studies have shown that the strongest risk factors for complaints of abnormal vaginal discharge and chronic fatigue are mental health-related factors—somatoform disorders and CMD.^32^,^33^ Thus, CMD are an integral component of our understanding of the aetiology of common reproductive complaints.

## TREATMENT OF COMMON MENTAL DISORDERS

The implications of the evidence linking CMD with social risk factors and other public health priorities is that if we can treat mental disorders effectively, we may find benefits not only to the patient's mental health, but to wider social and health outcomes as well.

Many developing countries have extremely meagre resources for mental illness and little progress has been made in improving treatment modalities. Although most countries have an essential drugs policy, about 20% do not even have the most commonly prescribed drugs for depression.^34^ In approximately half the countries of the world (all in the developing world), there is no more than one psychiatrist and one psychiatric nurse per 100,000 population; the numbers of psychologists and social workers working in mental health is even lower. As a result of this scarcity of mental health resources, the overwhelming majority of persons with depression would have little opportunity for specialist treatment. Thus, in the context of the considerable burden of CMD, the fact that the overwhelming majority of patients are only seen in primary care, and the great shortage of mental health specialists, treatments must be delivered in primary care or community settings by general or community health practitioners. Apart from being effective, such treatments must be affordable and accessible.

Until recently, all the evidence for effective treatment of depression was derived from randomized controlled trials in developed countries, and the cross-national applicability of these studies had been questioned on a number of grounds.^35^ These grounds include (i) cultural factors such as the local acceptability of specific interventions, (ii) health system factors such as the availability of human resources to implement intervention, (iii) costs and availability of medication, and (iv) individual patient factors such as pharmacodynamic variations among populations, all of which could influence the cross-cultural validity of treatment evidence. Three randomized controlled trials that studied the efficacy and cost-effectiveness of the treatment of depression in India, Uganda and Chile have been published.^17^,^36^,^37^ All these trials shared a number of features, including preparatory work in which measures for depression were translated and validated for the local culture and epidemiological studies were undertaken to estimate prevalence and risk factors. All the studies targeted poor populations. The Indian and Chilean trials were located in low-income, urban, primary or general healthcare settings while the Ugandan trial was in a poor rural community. All the studies tested treatment options that were intended to be feasible and affordable to the populations being studied.

So, what worked? All three trials had a psychological intervention; however, only the two trials that employed a group-based intervention found that this intervention was efficacious. The individual, psychological intervention used in India was no better than placebo; this lack of efficacy was, in all probability, due to the culturally unacceptable nature of a purely ‘talking’ intervention by a professional therapist. On the other hand, group therapy that emphasizes support and sharing among members of the same community was highly effective in Uganda and Chile. These group interventions were also part of a bottom–up approach in which people from the local area led the intervention. Antidepressants were used in two trials, one as a discrete treatment (in India), and one as part of a multimodal intervention along with group therapy (in Chile). The Indian trial demonstrated the superiority of antidepressants as compared to placebo, particularly in facilitating an early recovery. However, adherence to treatment declined rapidly after 2 months and this may have accounted for the absence of any significant effects at the 6 and 12 months' outcome. In the Chilean study, most patients in both groups received medication, the main difference being that the stepped-care group received appropriate doses for longer periods of time. Four factors might have influenced this: structured guidelines for medication, advocacy on behalf of the patient by the group leader when approaching the prescribing doctor, peer support, and empowerment of patients to take an active role to ensure that guidelines were enforced. The Ugandan trial employed no drug therapy at all. All trials had a measure of function or disability which showed significant improvements in the treatment group; the Indian trial showed that treating depression produces a significant reduction in total healthcare costs.

Thus, in all the three study sites, there was evidence for efficacy of depression interventions that were locally feasible and cost-effective among the poorest people in that setting. The associated improvement in function suggests benefits beyond mental health and beyond the individual who was treated, since improved function should benefit both the family and the community, and enable the person to cope better with social and economic difficulties. The studies demonstrate that some interventions found to be effective in developed countries were found effective at these study sites while others were not, perhaps due to local factors such as low adherence and lack of acceptability of specific treatments. Both elements suggest that it is worth trying interventions found to be effective in other cultures, but that their effectiveness needs to be tested when applied to new populations.

The studies also demonstrate that scientific evaluation of interventions, in the form of adequately powered randomized controlled trials, with relatively high response rates, are feasible in developing countries from both a practical and ethical viewpoint. While there continues to be a need for more studies among other populations to determine the cross-cultural applicability of these approaches, and to identify other interventions likely to be effective, the new evidence obliges physicians, policy-makers and donors to take action to reduce the burden of one of the most common and disabling illnesses in developing countries. Above all, it is time to use the new evidence to actively combat the scepticism of policy-makers that there is nothing that can be done for depression in developing countries.

## IMPLICATIONS FOR THEORY, POLICY AND RESEARCH

Evidence suggests that although the most consistent risk factors for CMD lie in the social and economic contexts of individuals' lives, both biological treatments (anti-depressants) and psychological treatments (group therapy) are efficacious and cost-effective. The apparent divergence of social origins and biological treatments for CMD has parallels with the multifactorial aetiological models well established for other chronic, non-communicable diseases such as diabetes mellitus. The key differences lie in the fact that pathophysiological processes in diseases such as diabetes are more clearly elucidated. Thus, the role of lifestyle and stressful events as well as the role of insulin resistance and genetic inheritance in the aetiology and prevention of diabetes are well-established. The existence of a social aetiology that triggers a biological pathological process in vulnerable individuals and which, despite the lack of direct action on the social aetiology, responds to biological treatments, is the basic principle of the theoretical rationale. The finding that antidepressants are efficacious is supportive evidence for a biological basis to the pathophysiology of CMD. Similarly, there is evidence that psychological treatments, such as cognitive–behavioural therapy, exert a therapeutic effect on depressive disorders, which are reflected in changes in brain metabolism.^38^ However, the precise mechanism through which social and biological factors interact to lead to CMD, or to enable recovery from CMD, remains unclear. I will now consider the implications of this evidence for policy and research.

### Policy

In societies where mental health services are poorly developed, it may be argued that preventive strategies aimed at strengthening protective factors in local communities may be a more sensible investment of scarce resources than duplicating the extensive mental healthcare systems of the developed world (whose existence has not led to any significant reduction in the prevalence of mental disorders). There is a potential for both primary and secondary preventive strategies.

In terms of *primary prevention*, two major themes which can be explored are education and economic empowerment. Although health policy has often considered mental health as a ‘luxury’ item when dealing with the health consequences of poverty, it is clear from the evidence presented that the poor are more likely to suffer from mental illness and tragic outcomes as a result of their illness.

In many developing countries, indebtedness to loan-sharks is a great source of stress and worry. Since the mid-1990s, the seasonal monsoon has consistently failed in some central regions of India leading to low harvests and, subsequently, lower incomes for farmers. The ones who have suffered the most have been the poorest subsistence farmers; those who were not credit-worthy enough to get bank loans and had to borrow money from loan-sharks at exorbitant rates of interest to tide over the financial crisis. With their crops failing, the farmers were faced with the stark choice of selling whatever few assets they still had or become bonded labour to the moneylender until the debt was repaid. It is not surprising, then, that these circumstances lead to severe mental distress and, ultimately, suicide.^39^ It is clear that here lies a potential preventive strategy in that local banks could step in and review their process of assessing credit-worthiness for persons who belong to the poorest sections of society. While there is no evidence specifically demonstrating the link between the access to micro-credit and suicide, many non-governmental organizations (NGOs) such as those run by Basix in India and the Bangladesh Rural Advancement Committee (BRAC) in Bangladesh are involved in setting up such loan facilities in rural areas. Provision of such loans may reduce mental illness by removing the key cause of stress: the threat posed by the informal moneylender. The NGO programmes for poverty alleviation target not only credit facilities, but also gender equity, basic healthcare, nutrition, education and human rights issues. An evaluation of the BRAC poverty alleviation programmes, which reach out to millions of the poorest people in Bangladesh, indicates that the psychological well-being of women who are BRAC members is better than those who are not.^40^

The key to *secondary prevention* is placing mental illness, in particular CMD, onto the priority agenda for primary healthcare by local policy-makers. There is a need to move the subject of CMD from its current home within the isolated and marginalized realm of psychiatry into the broader, community-oriented public health arena. Thus, greater emphasis is required on developing innovative methods of training general health workers to recognize and effectively treat CMD. The author has written *Where there is no psychiatrist*, a healthcare manual modelled along the lines of the classic manual, *Where there is no doctor*, which follows these principles.^41^ The manual is now being translated into Indian languages by the Voluntary Health Association of India, New Delhi.

### Research

Despite the compelling evidence of an association between CMD and economic deprivation, it is important to recognize that the majority of people living even in squalid poverty remain well, cope with the daily grind of existence and do not succumb to the stressors they face in their lives. Indeed, this is the real challenge for public health researchers, i.e. to identify the protective and nurturing qualities in those who do not become depressed when faced with difficult economic circumstances, for therein lies a potential to help and prevent mental health problems. Although we now have enough evidence from efficacy trials to guide our choices for specific treatments for CMD, we still do not have a model through which these can be integrated into routine primary care in an effective and affordable manner. A combination of an antidepressant with a psychosocial intervention, providing more resource-intensive interventions according to individual patient needs (the stepped-care model) may be the ideal way of improving clinical outcomes in patients with CMD in routine primary care. Recent trials reporting the efficacy of a collaborative management protocol may provide a useful route to improving outcomes.^42^ An innovative intervention which combines the principles of stepped-care and collaborative care will be evaluated in a new cluster randomized trial to be implemented in Goa, India over the next few years. Finally, there is the need for strong collaboration between biological and epidemiological psychiatric research to uncover the precise pathways through which social factors lead to biological changes, which lie at the heart of the distress and treatment of CMD.

## References

[1] Goldberg D, Huxley P (1992). Common mental disorders: A biosocial model.

[2] World Health Organization (WHO) (1992). The ICD-10 classification of mental and behavioural disorders.

[3] Jacob KS, Everitt BS, Patel V (1998). The comparison of latent variable models of nonpsychotic psychiatric morbidity in four culturally different populations. Psychol Med.

[4] Lewis G (1992). Dimensions of neurosis. Psychol Med.

[5] Tyrer P (2001). The case for cothymia: Mixed anxiety and depression as a single diagnosis. Br J Psychiatry.

[6] WHO (2001). The World health report 2001: Mental health: New understanding, new hope.

[7] Chisholm D, Sekar K, Kumar K (2000). Integration of mental health care into primary care. Demonstration cost-outcome study in India and Pakistan. Br J Psychiatry.

[8] Patel V, Kleinman A (2003). Poverty and common mental disorders in developing countries. Bull World Health Organ.

[9] WHO (1995). Mental illness in general health care: An international study.

[10] Patel V (1999). The epidemiology of common mental disorders in South Asia. NIMHANS J.

[11] Patel V, Pereira J, Coutinho L (1998). Poverty, psychological disorder and disability in primary care attenders in Goa, India. Br J Psychiatry.

[12] Patel V, Todd CH, Winston M (1998). The outcome of common mental disorders in Harare, Zimbabwe. Br J Psychiatry.

[13] Patel V, Pereira J, Mann A (1998). Somatic and psychological models of common mental disorders in India. Psychol Med.

[14] Patel V, Gwanzura F, Simunyu E (1995). The explanatory models and phenomenology of common mental disorder in Harare, Zimbabwe. Psychol Med.

[15] Patel V, Pereira J, Coutinho L (1997). Is the labelling of common mental disorders as psychiatric illness useful in primary care?. Indian J Psychiatry.

[16] Linden M, Lecrubier Y, Bellantuono C (1999). The prescribing of psychotropic drugs by primary care physicians: An international collaborative study. J Clin Psychopharmacol.

[17] Patel V, Chisholm D, Rabe–Hesketh S (2003). The efficacy and cost-effectiveness of a drug and psychological treatment for common mental disorders in general health care in Goa, India: A randomised controlled trial. Lancet.

[18] Patel V, Araya R, Lima MS (1999). Women, poverty and common mental disorders in four restructuring societies. Soc Sci Med.

[19] Piccinelli M, Wilkinson G (2000). Gender differences in depression. Critical review. Br J Psychiatry.

[20] Patel V, Kirkwood BR, Pednekar S Gender disadvantage and reproductive health risk factors for common mental disorder in women: A community survey in India. Arch Gen Psychiatry (in press).

[21] Chandran M, Tharyan P, Muliyil J (2002). Post-partum depression in a cohort of women from a rural area of Tamil Nadu, India. Incidence and risk factors. Br J Psychiatry.

[22] Cooper P, Tomlinson M, Swartz L (1999). Post-partum depression and the mother–infant relationship in a South African peri-urban settlement. Br J Psychiatry.

[23] Patel V, Rodrigues M, De Souza N (2002). Gender, poverty and post-natal depression: A cohort study from Goa, India. Am J Psychiatry.

[24] Oates M (2003). Suicide: The leading cause of maternal death. Br J Psychiatry.

[25] Aaron R, Joseph A, Abraham S (2004). Suicides in young people in rural southern India. Lancet.

[26] Phillips MR, Li X, Zhang Y (2002). Suicide rates in China, 1995–99. Lancet.

[27] Patel V, Rahman A, Jacob KS (2004). Effect of maternal mental health on infant growth in low income countries: New evidence from South Asia. BMJ.

[28] Rahman A, Iqbal Z, Bunn J (2004). Impact of maternal depression on infant nutritional status and illness: A cohort study. Arch Gen Psychiatry.

[29] Koenig M, Jejeebhoy S, Singh S (1998). Investigating women's gynaecological morbidity in India: Not just another KAP survey. Reproductive Health Matters.

[30] Hawkes S, Morison L, Foster S (1999). Managing reproductive tract infections in women in low-income, low-prevalence situations: An evaluation of syndromic management in Matlab, Bangladesh. Lancet.

[31] Patel V, Oomman NM (1999). Mental health matters too: Gynecological morbidity and depression in South Asia. Reproductive Health Matters.

[32] Patel V, Kirkwood BR, Weiss H (2005). Chronic fatigue in developing countries: Population based survey of women in India. BMJ.

[33] Patel V, Pednekar S, Weiss H Why do women complain of vaginal discharge? A population survey of infectious and pyschosocial risk factors in a South Asian community. Int J Epidemiol 2005 (in press).

[34] WHO (2001). Atlas country profiles of mental health resources.

[35] Patel V (2000). The need for treatment evidence for common mental disorders in developing countries. Psychol Med.

[36] Araya R, Rojas G, Fritsch R (2003). Treating depression in primary care in low-income women in Santiago, Chile: A randomised controlled trial. Lancet.

[37] Bolton P, Bass J, Neugebauer R (2003). Group interpersonal psychotherapy for depression in rural Uganda. JAMA.

[38] Goldapple K, Segal Z, Garson C (2004). Modulation of cortical–limbic pathways in major depression: Treatment-specific effects of cognitive behavior therapy. Arch Gen Psychiatry.

[39] Sundar M (1999). Suicide in farmers in India. Br J Psychiatry.

[40] Chowdhury A, Bhuiya A, Leon D, Walt G (2001). Do poverty alleviation programs reduce inequities in health? The Bangladesh experience. Poventy, inequality and health.

[41] Patel V (2003). Where there is no psychiatrist.

[42] Katon W, Von Korff M, Lin E (1995). Collaborative management to achieve treatment guidelines: Impact on depression in primary care. JAMA.

